# Influence of Forest Management Regimes on Forest Dynamics in the Upstream Region of the Hun River in Northeastern China

**DOI:** 10.1371/journal.pone.0039058

**Published:** 2012-06-18

**Authors:** Jing Yao, Xingyuan He, Anzhi Wang, Wei Chen, Xiaoyu Li, Bernard J. Lewis, Xiaotao Lv

**Affiliations:** 1 State Key Laboratory of Forest and Soil Ecology, Institute of Applied Ecology, Chinese Academy of Sciences, Shenyang, People’s Republic of China; 2 Graduate University of Chinese Academy of Sciences, Beijing, People’s Republic of China; DOE Pacific Northwest National Laboratory, United States of America

## Abstract

Balancing forest harvesting and restoration is critical for forest ecosystem management. In this study, we used LANDIS, a spatially explicit forest landscape model, to evaluate the effects of 21 alternative forest management initiatives which were drafted for forests in the upstream region of the Hun River in northeastern China. These management initiatives included a wide range of planting and harvest intensities for *Pinus koraiensis*, the historically dominant tree species in the region. Multivariate analysis of variance, Shannon's Diversity Index, and planting efficiency (which indicates how many cells of the target species at the final year benefit from per-cell of the planting trees) estimates were used as indicators to analyze the effects of planting and harvesting regimes on forests in the region. The results showed that the following: (1) Increased planting intensity, although augmenting the coverage of *P. koraiensis*, was accompanied by decreases in planting efficiency and forest diversity. (2) While selective harvesting could increase forest diversity, the abrupt increase of early succession species accompanying this method merits attention. (3) Stimulating rapid forest succession may not be a good management strategy, since the climax species would crowd out other species which are likely more adapted to future climatic conditions in the long run. In light of the above, we suggest a combination of 30% planting intensity with selective harvesting of 50% and 70% of primary and secondary timber species, respectively, as the most effective management regime in this area. In the long run this would accelerate the ultimate dominance of *P. koraiensis* in the forest via a more effective rate of planting, while maintaining a higher degree of forest diversity. These results are particularly useful for forest managers constrained by limited financial and labor resources who must deal with conflicts between forest harvesting and restoration.

## Introduction

Conservation and responsible utilization are two essential facets of the harmonious relationship between humans and nature, as well as two interrelated strategies of sustainable forest development. Forests around the world are shrinking due to over-exploitation [1]–[3]. Because forests play a critical role in water conservation [4], [5], prevention of soil erosion [6], [7] and climate regulation [8], ecological problems such as massive soil erosion, catastrophic flooding, and severe dust storms often follow forest degradation. As a reaction to this, a host of theories and methods on forest restoration have been explored by researchers [9]–[11]. Still, some research has questioned whether the strategy to restore forest ecosystems following the pathway of historical forest succession is a proper one under conditions of uncertain variation of climatic patterns induced by climate change [12], [13]. Ravenscroft et al. [14] have also pointed out that high diversity of conditions and species within forest landscapes is the most effective means of ensuring the future resistance of ecosystems to climate-induced declines in productivity.

Meanwhile, some research on the effects of utilization especially timber harvesting, on forest ecosystems has found that while there is an initial setback of forest succession after the forest is harvested, harvesting can increase ecosystem diversity [15]–[17]. Yet relatively little research has explored how to ensure a balance between restoration and harvesting [18], which is important in most regions where forest industry is dominant.

Due to rapid population growth, coupled with agricultural development, urban construction, and unsound forest management, the degradation of forest resources in China, particularly in the North, has been accelerating, with the resultant deterioration of the environment [3]. To prevent this, the Three-North (which includes northwestern, north and northeastern of China) Shelterbelt project (http://www.forestry.gov.cn) was launched in 1978, aiming to prevent soil erosion and desertification by increasing forest coverage through afforestation and protection of farmland, as well as enhance urban eco-security in North China.

In the Northeast, forests have been degraded due to unsound timber harvest and farming [3], [19]. The forest in the upstream area of the Hun River (UHR) in Qingyuan county of Liaoning province is an important focus of the Three-North Shelterbelt Project, since it benefits the eco-environment of the downstream area of Hun River (DHR), a region of heavy industry and the population center of Northeast China. Unfortunately, the forests in the UHR were over-exploited in the last century and degraded from mixed broad-leaved *P. koraiensis* forest [20] to secondary forests dominated by *Quercus. mongolica*, *Betula spp.*, *Populus spp.* and other early or mid-successional species. Thus forests in the UHR are in a transitional stage from early- to mid-succession and accelerating the successional process in this region is urgent for ecological security of the urban centers of the DHR. At the same time, forest industry is still one of the basic industries in Qingyuan county, just as in other forested areas of northeast China. In accordance with the Three-North Shelterbelt project and the needs of local forest industry, half of the forest in Qingyuan county is reserved as public benefit forest where timber harvesting is forbidden; while the remaining half serves as a timber resource.

Given the above context, there are two key points of concern in this article: (1) the choice of planting intensity for accelerating the successional path toward the climax forest; and (2) at the landscapes scale, whether the harvest in forests designated for timber harvesting would contribute to the degradation of the overall forest ecosystem.

LANDIS is a powerful tool for evaluating alternative forest management strategies at a landscape scale [21]–[25] due to its ability to simulate forest variation at large spatial and temporal scales, a capability beyond the limits of traditional studies [22], [26], [27]. In the past decade, LANDIS has been used by researchers across North America, Europe and China [28] as a tool for decision making in forest management. For example, Wang et al. [27] examined effects of different planting densities on forest restoration, providing a guide for forest management decisions which has proven to be one of the most cost-effective and least labor-intensive in North China. Cairns et al. [23] considered alternative restoration strategies for insect-affected landscapes by LANDIS and suggested that it is necessary to consider the patterns of hosts on the landscape as well as the landscape composition.

In this research, we utilized LANDIS to examine the effects of different planting regimes and harvest regimes on forest succession to explore the balance between restoration and harvesting. There are 3 specific questions we address in this paper. (1) Which planting intensity is the most effective for restoring the forest ecosystem while taking diversity and limited financial and labor resources into account? (2) How does harvesting affect forest dynamics – i.e., does harvesting increase biodiversity of the landscape and which harvest intensity should be selected? (3) How do individual species respond to forest management regimes which involve different planting and harvest intensities?

## Methods

### 1 Study Area

**Figure 1:**
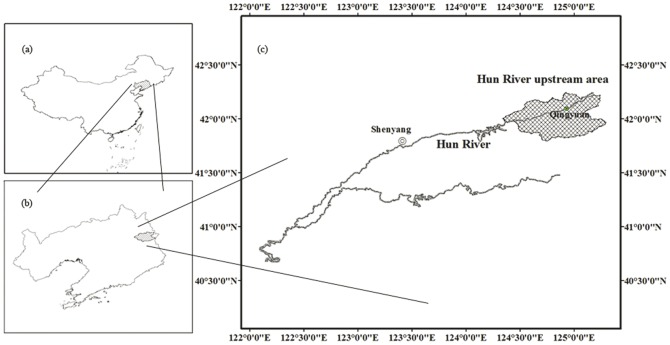
Location of the study area. a– Liaoning province in Northeast China; b– Qingyuan County in Liaoning province; and c– Hun River upstream area.

The upper Hun River area extends from 41°47'52'' ∼ 42°28'25''N,124°20'06'' ∼ 125°28'58''E (Fig. 1), and is characterized by a temperate continental monsoon climate. The mean annual temperature is 6.6°C and mean annual precipitation is 788 mm (data provided by the Qingyuan County Forestry Bureau). The average altitude of this region is 470 m, ranging from 150 m to 1086 m. The total study area encompasses 2.5×10^5^ ha. The forest in this area was at one time the climax community of the *P. koraiensis* and deciduous broad-leaved mixed forest. However, the natural forest was severely affected by human disturbance in the early 20th century. It has now been replaced by a secondary mixed forest which includes *P. koraiensis*, *Q. mongolica*, *Larix olgensis*, *Pinus tabulaeformis*, *Pinus densiflora*, *Pinus sylvestris var. mongolica*, *Fraxinus rhynchophylla*, *Fraxinus chinensis*, *Juglans mandshurica*, *Betula platyphylla*, *Populus davidiana*, *Acer pictum subsp. mono*, *Ulmus pumila*, *Tilia amuresis*, *Abies nephrolepis*, *Picea asperata* and other lesser species.

### 2 Description of LANDIS

LANDIS is a spatially explicit, stochastic, raster-based landscape model which facilitates the study of the effects of natural and anthropogenic disturbances, vegetational succession, management strategies and their interactive effects on forest landscapes [22], [26], [29]. It simulates species-level forest dynamics by tracking the presence or absence of species age cohorts at 10-year time steps under natural and anthropogenic disturbances, including fire, wind throw, insects and disease, harvesting, and fuel management. It simulates forest landscape change over large spatial (10^3^–10^7 ^ha) and temporal (10^1^–10^3^ years) scales with flexible resolutions (10–500 m pixel size). The model is described in detail elsewhere [25], [26], [30]. The version used in this research is LANDIS 6.0.

#### 2.1 Parameterization of LANDIS

There are two types of inputs in LANDIS: necessary inputs and optimal inputs. The necessary inputs include species life history attributes, species composition maps with associated presence/absence and age information for species, land type maps and species establishment coefficients for each land type. The optimal inputs include disturbance and management parameters including harvesting, planting and fire.

#### 2.2 Species attributes and species composition maps

Sixteen common tree species in the study area are included in our LANDIS simulation (Table 1). Species life history attributes were derived from the literature on species characteristics in this region [31]–[33], the parameterization of other research on northeastern China, and consultations with local experts [22], [34], [35]. The species composition map was derived from an extant stand map of 2006 and a stand attribute database, the latter of which was a component of 2006 forest inventory data provided by the Qingyuan County Forestry Bureau. The forest stand map recorded the boundaries of stands. The stand attribute database provided information on the relative percentage of canopy species, the average age of dominant canopy species, timber production, and crown density. The forest composition map was processed at a resolution of 60 m×60 m, which yielded 1320 rows ×836 columns. Each cell contained the presence/absence and age cohorts of all of the 16 tree species. For each cell in a stand, a stand-based assignation (SBA) approach [36] was used to stochastically assign species age cohorts to that cell based on forest inventory data.

**Table 1:** Species’ life attributes for forests in the upstream area of the Hun River in northeastern China.

Species	LONG	MTR	ST	FT	ED	MD	VP	MVP
*Pinus koraiensis*	400	40	5	1	50	200	0	0
*Pinus tabulaeformis*	200	30	2	1	100	500	0	0
*Pinus densiflora*	200	30	2	1	100	500	0	0
*Pinus sylvestris var. mongolica*	250	40	2	2	30	100	0	0
*Larix olgensis*	300	30	1	5	100	400	0	0
*Picea asperata*	300	30	5	3	80	150	0	0
*Abies nephrolepis*	250	40	5	3	80	150	0	0
*Populus davidiana*	100	8	1	2	−1	−1	1	10
*Betula platyphylla*	150	15	1	1	200	4000	0.8	50
*Ulmus pumila*	250	10	2	4	300	1000	0.3	60
*Fraxinus chinensis*	250	30	3	3	50	150	0.3	80
*Fraxinus rhynchophylla*	250	30	3	3	50	150	0.3	80
*Juglans mandshurica*	250	15	3	4	50	150	0.9	60
*Quercus mongolica*	350	40	3	5	20	200	0.9	60
*Acer pictum subsp. mono*	250	10	4	2	120	350	0.3	50
*Tilia amuresis*	300	30	4	4	50	100	0.9	30

Long– longevity (years); MTR–age of maturity (years); ST-shade tolerance class; FT–fire tolerance class; ED–effective seeding distance (m); MD–maximum seeding distance (m); VP–vegetative reproduction probability; MVP–minimum age of vegetative reproduction (years).

#### 2.3 Land type map

**Figure 2:**
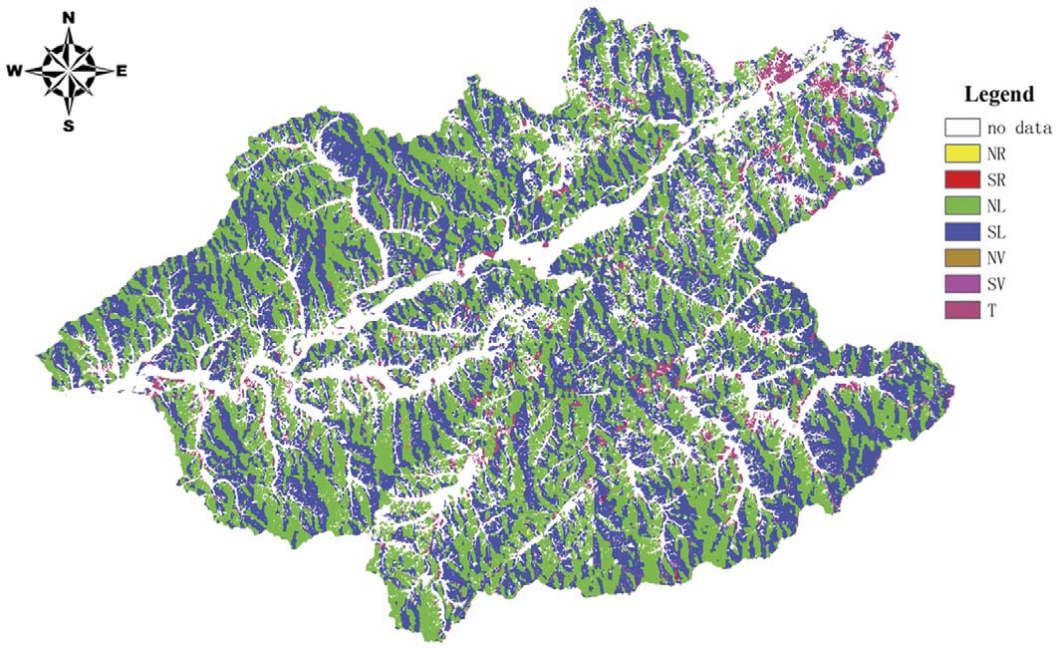
Landtype map of the Hun River upstream area. NR–North Ridge; SR–South Ridge; NL–North Slope; SL–South Slope; NV–North Slope of valley; SV–South Slope of valley; and T–terrace.

In LANDIS the heterogeneous landscape is stratified into relatively homogeneous units (land types or eco-regions) in LANDIS. Within each land type, environments for species establishment are assumed to be similar [26]. In this study, we first extracted the water body and city out of the land type map. Then we derived seven land types (Fig. 2), primarily based on terrain attributes of the 2006 forest inventory data and the 1992 Digital Elevation Model (DEM) of Qingyuan County at a resolution of 30 m×30 m which was downloaded from http://www1.csdb.cn/. Seven kinds of terrain were delineated: North Ridge (NR), South Ridge (SR), North Slope (NL), South Slope (SL), North Slope of valley (NV), South Slope of valley (SV) and terrace (T). There were no non-active land types in our land type map. The seven active land types accounted for 0.04%, 0.04%, 50.38%, 44.19%, 0.13%, 0.37% and 4.85% of the total area, respectively.

The species establishment coefficients of a land type are critical. They estimate the probability of a species successfully establishing on that land type, given the environmental conditions encapsulated by that type. We estimated the species establishment coefficients of land types from the literature on species characteristics in this region [31]–[33] and from parameterizations in other research on northeastern China [22], [34], [35].

### 3 Simulation Scenarios

The initial forest composition and land type maps including species/age classes realistically represented the status of the forests in the study area in 2006. We simulated 21 scenarios, including the natural succession process without planting and harvest, five levels of planting intensity and fifteen different combinations of the five planting intensity levels and three selective harvest intensity levels (Table 2). *P. koraiensis* was planted under broadleaved trees that were >9 years old and whose canopies were broad enough to provide a shaded environment for the seedlings of *P. koraiensis* [37]. Harvest age of species in this study followed National Forest Resources Continuous Inventory Technique Formula (Table 3). Three replicates of each scenario were simulated. All scenarios were simulated up to 300 years to examine the long-term effects of planting intensity and harvest intensity on forest succession. The study area was divided into 10 management areas (MA) identified via planting and harvest options practiced there: (1) short-rotation timber of broadleaved trees which are >9 years old (MA 1); (2) short-rotation timber of all trees other than those included in MA 1 (MA 2); (3) fast-growing timber of broadleaved trees which are >9 years old (MA 3); (4) fast-growing timber of all trees except those included in MA 3 (MA 4); (5) public forest of broadleaved trees that are >9 years old (MA 5); (6) public forest of all trees except those included in MA 5 (MA 6); (7) general natural timber of broadleaved trees that are >9 years old (MA 7); (8) general natural timber of all trees except those included in MA 7 (MA 8); (9) general plantation timber of broadleaved trees that are >9 years old (MA9); and (10) general plantation timber of all trees except those included in MA 9 (MA 10). The harvest regimes were implemented on all management areas except MA5 and MA6, where harvesting of public forests is forbidden according to the Three-North Shelterbelt project.

**Table 2:** The scenarios simulated by LANDIS 6.0.

Scenario	Planting intensityof *P. koreaiensis*	Selective Harvest(general timber forest)	Selective Harvest (short-rotation forestand fast-growing forest)	
N	–	–	–	
P1	5%	–	–	
P2	10%	–	–	
P3	30%	–	–	
P4	50%	–	–	
P5	70%	–	–	
P1H1	5%	10%	30%	
P1H2	5%	30%	50%	
P1H3	5%	50%	70%	
P2H1	10%	10%	30%	
P2H2	10%	30%	50%	
P2H3	10%	50%	70%	
P3H1	30%	10%	30%	
P3H2	30%	30%	50%	
P3H3	30%	50%	70%	
P4H1	50%	10%	30%	
P4H2	50%	30%	50%	
P4H3	50%	50%	70%	
P5H1	70%	10%	30%	
P5H2	70%	30%	50%	
P5H3	70%	50%	70%	

Note: *P. koraiensis* was planted under broadleaved trees which were >9 years old, whose canopies were broad enough to provide a shaded environment for the seedlings of *P. koraiensis* [37].

**Table 3:** Harvest age (years) of species in this study according to National Forest Resources Continuous Inventory Technique Formula (China).

Species	SRT	FGT	GPT	GNT
*Pinus koraiensis*	>40	–	>80	>120
*Pinus tabulaeformis*	–	–	>40	>60
*Pinus densiflora*	–	–	>40	>100
*Pinus sylvestris var. mongolica*	>20	–	>40	>100
*Larix olgensis*	>20	>20	>40	>100
*Picea asperata*	–	–	>80	>120
*Abies nephrolepis*	–	–	>40	>100
*Populus davidiana*	>10	>20	>20	>20
*Betula platyphylla*	>10	>20	>40	>60
*Ulmus pumila*	–	–	>40	>60
*Fraxinus chinensis*	>20	–	>50	>80
*Fraxinus rhynchophylla*	>20	–	>50	>80
*Juglans mandshurica*	>20	–	>50	>80
*Quercus mongolica*	–	–	>50	>80
*Acer pictum subsp. mono*	>30	–	>50	>80
*Tilia amuresis*	–	–	>50	>80

SRT– short-rotation timber; FGT– fast-growing timber; GPT– general plantation timber; GNT– general natural timber.

### 4 Analysis Methods

Utilizing SPSS 18.0, the area percentage (AP) of each species was calculated from the output map for each 10-year step in the LANDIS output statistical program to depict the trend for each species over the 300 simulated years.

We analyzed the AP of each species utilizing multivariate analysis of variance (MANOVA) with planting intensity and harvest intensity in SPSS 18.0. Pillai’s Trace statistic was used to test the hypotheses that planting intensity and harvest intensity affect the area of species in the study area, because it is the least sensitive of the four multivariate tests provided by SPSS with respect to the heterogeneity of variance assumption of MANOVA [27]. Shannon’s Diversity Index of each scenario in the 300^th^ year was calculated in FRAGSTATS 3.0. Then we analyzed differences in Shannon’s Diversity values induced by different harvest intensities using one-way ANOVA in SPSS 18.0. The LSD was used to test the hypotheses that different harvest intensities induce differences in Shannon’s Diversity values.

We also developed and calculated planting efficiency to test the response of *P. koraiensis* coverage to different planting intensities via the following formula: , where PE is planting efficiency; A_i_ is the area (cell) of *P. koraiensis* coverage at year 300 under different planting intensity scenarios; A_N_ is the area (cell) of *P. koraiensis* coverage at year 300 in the natural succession scenario without any planting and harvesting; and A_j_ is the overall planting area (cell) in different planting scenarios. Because planting is a way of restoration to increase the seed source [38], the PE indicates how many cells of the target species at year 300 benefit from per-cell of planting *P. koraiensis* under the different planting intensities.

## Results

**Figure 3:**
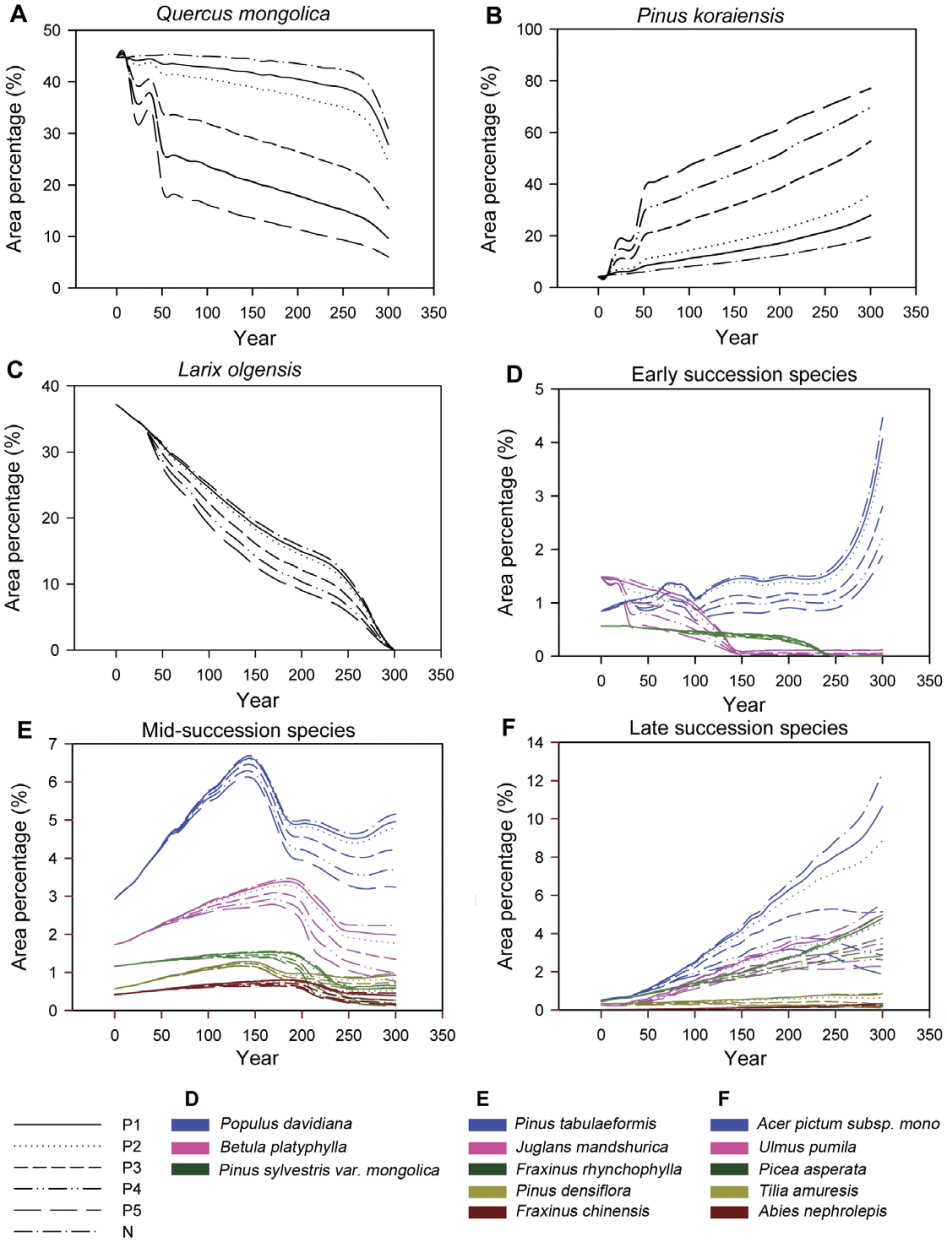
Response of area proportion of different species to different planting intensities. P1–P5 Definitions are given in Table 2.

In the N scenario (Table 2), in which there was no planting or harvesting, mid- and late-succession species (*P. koraiensis*, *P. asperata, A. nephrolepi, U. pumila, A. pictum subsp. mono and T. amuresis)* showed increasing trends in percentage of total area (Fig.3 F), whereas early succession species (*P. sylvestris var. mongolica*, *L. olgensis* and *B. platyphylla*) showed decreasing trends (Fig.3 D). *Q. mongolica*, which is a mid-succession species, also demonstrated decreasing trends in area percentage (Fig.3 A). Trends for *P. tabulaeformis*, *P. densiflora*, *F. chinensis*, *F. rhynchophylla and J. mandshurica*, which are mid-tolerant species, first increased and then decreased (Fig.3 E). The area percentage of *P. koraiensis* was 4.16% in the first year and rose to 19.53% by year 300. The area percentages of *Q. mongolica* and *L. olgensis* were 44.69% and 37.18%, respectively, in the initial year, and fell to 30.94% and 0%, respectively, by year 300. Although *Q. mongolica* was still the most abundant species in the study area at year 300, the composition of the Hun River upstream forest changed from one dominated by *Q. mongolica* and *L. olgensis* to one dominated by *Q. mongolica* and *P. koraiensis.* Thus the successional trajectory of the forest was slowly heading toward the climax forest in this region.

**Figure 4:**
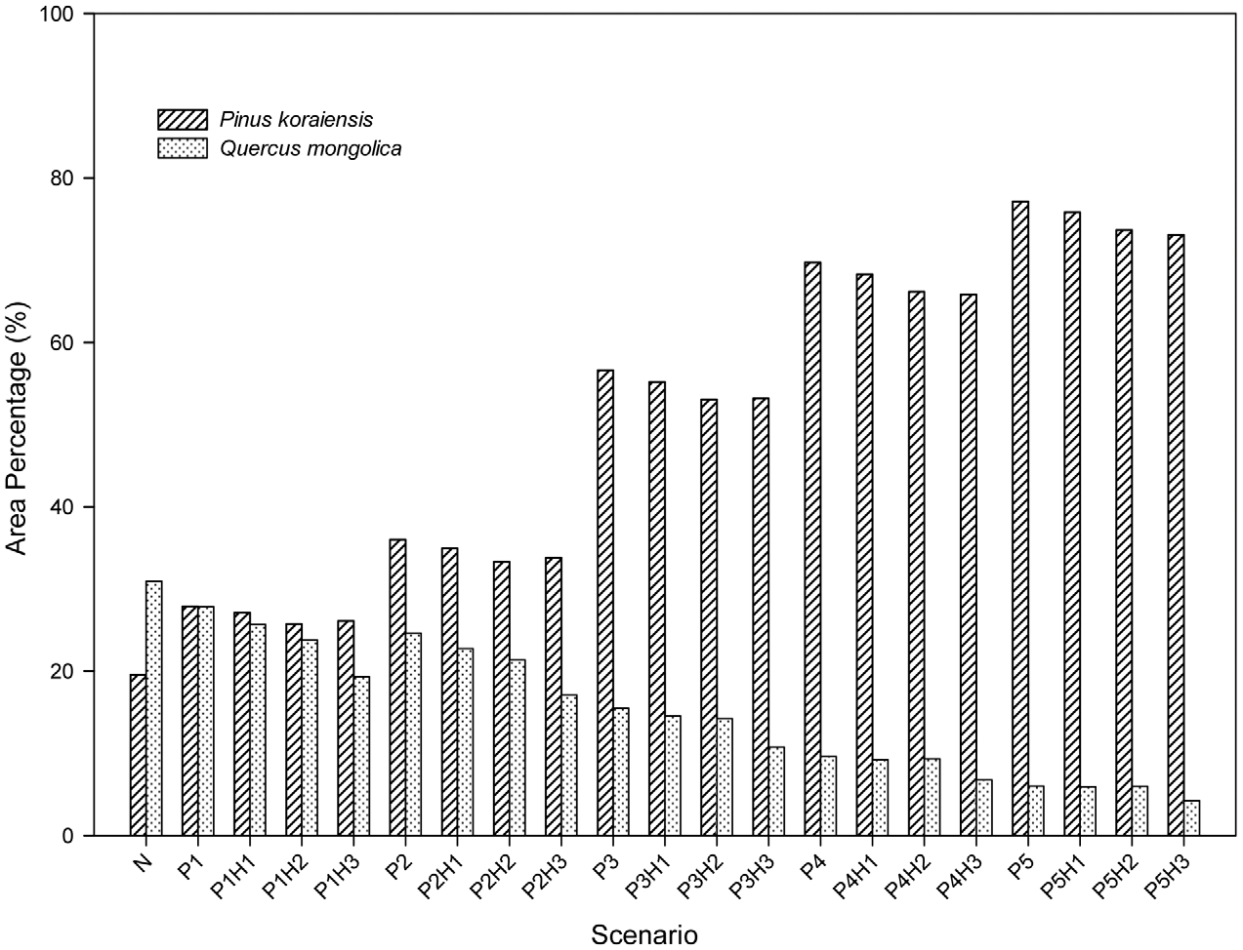
Area percentage of *Pinus koraiensis* and *Quercus mongolica* in simulated scenarios at year 300. The definitions of the scenarios may be found in Table 2.

In the five P scenarios (Table 2) in which there were planting, most species displayed the same area percentage trends as they did in the N scenarios (Fig. 3). Nevertheless, the planting of *P. koraiensis* did suppress the increasing trends of other species (Fig. 3) and accelerate the successional process toward a climax forest dominated by *P. koraiensis* (Fig. 3 B). Increasing trends for *A. pictum subsp. mono*, *U. pumila*, *T. amuresis* and *A. nephrolepis* were either diminished or reversed (Fig. 3 F). At year 300, the area percentage of *P. koraiensis* was nearly same as that of *Q. mongolica* under the 5% planting intensity scenario and reached 56% under the 30% planting intensity scenario (Fig. 4). Although the area percentage of *P. koraiensis* at year 300 increased with increasing planting intensity, the sensitivity of response of *P. koraiensis* to planting intensity decreased with increasing intensity (Table 4).

**Table 4:** Planting yield of different planting intensity in the 300th year of our simulation.

Scenario	PlantingIntensity (%)	Area Percentageat Year 300 (%)	Area PercentageIncrease (%)	Planting Efficiency
**P1**	5	27.87	8.34	3.60
**P2**	10	35.99	16.46	3.54
**P3**	30	56.63	37.10	2.67
**P4**	50	69.76	50.23	2.17
**P5**	70	77.14	57.61	1.77

The definition of the scenarios is in Table 2 of this paper.

The definition of Planting Efficiency is in the section 2.4 of this paper.

**Figure 5:**
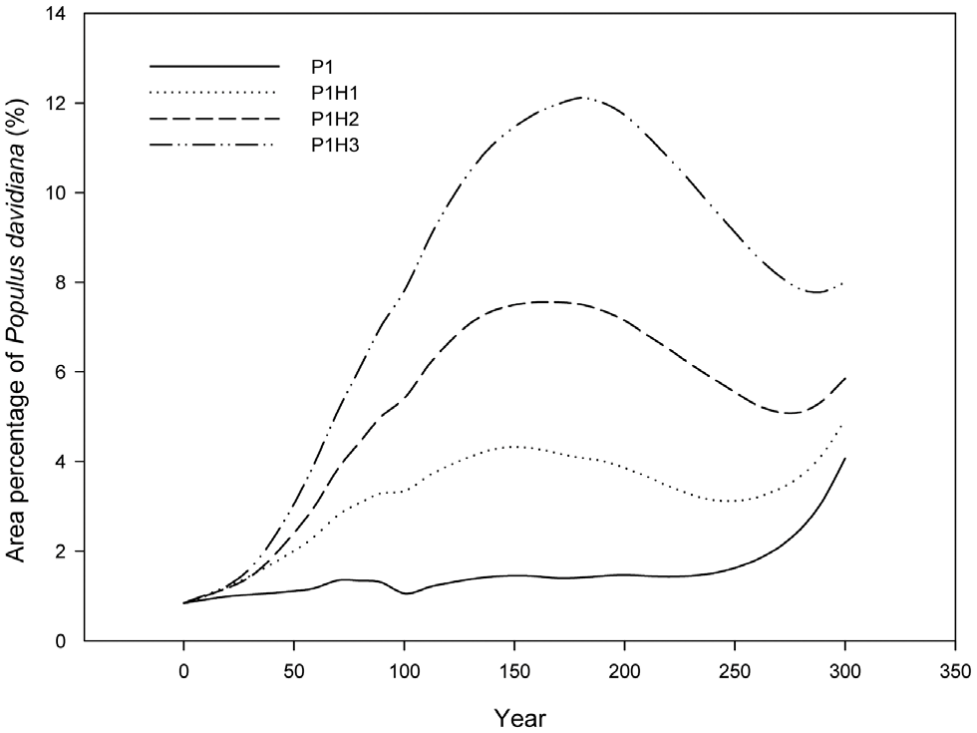
Response of area percentage of *Populus spp.* to different harvest intensities. P1, P1H1, P1H2, P1H3: Definitions may be found in Table 2.

In the PH scenarios (Table 2), in which there was both planting and harvesting, trends in area percentage of species (not shown in the paper) were similar to those in the P scenarios, except for *P. davidiana*, as exemplified by comparing values for 5% planting intensity with those for combinations of 5% planting intensity and different harvest regimes (Fig. 5). The dynamic of area percentage for *P. davidiana* was stable until there was an abrupt increase at year 250 under 5% planting intensity scenario without harvesting. However, a similar increase occurred around year 50 under the PH scenarios.

The test of MANOVA on PH scenarios showed that both P regimes and H regimes had significant effects on the dynamics of the forest in the study area (Table 5). For individual species, P regimes had significant effects on most species, with the exception of *L. olgensis* and *P. sylvestris var. mongolica;* while H regimes only had significant effects on *Q. mongolica*, *L. olgensis*, *P. davidiana*, *B. platyphylla* and *U. pumila* (Table 5). For more detail, the effects on species between different harvest levels were tested by the custom hypothesis test (simple contrast, the H1 which is referenced to Table 2 was chosen as the reference) and showed that increasing harvest intensity had a significant positive effect on *P. davidiana*, *B. platyphylla*, *P. densiflora*, *U. pumila* and *T. amuresis* and a negative effect on *L. olgensis* and *Q. mongolica* (Table 6).

**Table 5:** MANOVA and ANOVA results for area proportion of species of our study area as a function of planting intensity and harvest intensity.

Effect	Planting				Harvest			
	Pillai’s trace/type III SS	df	F	P	Pillai’s trace/type III SS	df	F	P
MANOVA test	1.977	68	26.648	<0.001	1.122	34	32.355	<0.001
ANOVA test								
*Pinus koraiensis*	85378.164	4	111.771	<0.001	42.035	2	0.110	0.896
*Pinus tabulaeformis*	45.402	4	12.872	<0.001	1.699	2	0.963	0.382
*Pinus densiflora*	1.817	4	13.381	<0.001	0.194	2	2.851	0.059
*Pinus sylvestris var. mongolica*	0.038	4	0.239	0.916	0.038	2	0.476	0.621
*Larix olgensis*	939.107	4	1.965	0.099	1401.289	2	5.865	0.003
*Picea asperata*	20.663	4	4.146	<0.001	0.196	2	0.078	0.925
*Abies nephrolepis*	0.093	4	5.361	<0.001	0.009	2	1.018	0.362
*Populus davidiana*	289.569	4	17.732	<0.001	1161.638	2	142.270	<0.001
*Betula platyphylla*	8.837	4	14.670	<0.001	1.659	2	5.508	0.004
*Ulmus pumila*	177.853	4	13.394	<0.001	71.537	2	10.775	<0.001
*Fraxinus chinensis*	1.651	4	23.867	<0.001	0.016	2	0.459	0.632
*Fraxinus rhynchophylla*	5.990	4	9.878	<0.001	0.010	2	0.034	0.966
*Juglans mandshurica*	41.342	4	33.690	<0.001	0.618	2	1.006	0.367
*Quercus mongolica*	26094.143	4	91.088	<0.001	877.550	2	6.127	0.002
*Acer pictum subsp. mono*	554.685	4	29.920	<0.001	0.366	2	0.039	0.961
*Tilia amuresis*	7.453	4	161.711	<0.001	0.070	2	3.051	0.048

**Table 6:** Contrast estimate of effects of different harvest intensity on area percentage of species for individual species.

Dependent variables	Level 2 vs. level 1		Level 3 vs. level 1	
	ContrastEstimate	P	ContrastEstimate	P
*Pinus koraiensis*	−0.627	0.690	−0.648	0.680
*Pinus tabulaeformis*	0.065	0.545	0.148	0.167
*Pinus densiflora*	0.150	0.467	0.049	0.020
*Pinus sylvestris var. mongolica*	−0.020	0.366	−0.017	0.442
*Larix olgensis*	−2.127	0.087	−4.252	0.001
*Picea asperata*	0.008	0.949	0.047	0.711
*Abies nephrolepis*	6.452E–5	0.993	−0.009	0.219
*Populus davidiana*	1.548	<0.001	3.847	<0.001
*Betula platyphylla*	0.054	0.220	0.145	0.001
*Ulmus pumila*	0.493	0.018	0.961	<0.001
*Fraxinus chinensis*	0.003	0.819	0.014	0.358
*Fraxinus rhynchophylla*	−0.003	0.942	0.008	0.857
*Juglans mandshurica*	0.022	0.723	0.086	0.172
*Quercus mongolica*	−0.567	0.555	−3.156	0.001
*Acer pictum subsp. mono*	0.053	0.830	0.065	0.792
*Tilia amuresis*	0.004	0.771	0.028	0.024

Level1: selectively harvesting 10% of general timber forest and 30% of other timber forest

Level2: selectively harvesting 30% of general timber forest and 50% of other timber forest.

Level3: selectively harvesting 50% of general timber forest and 70% of other timber forest.

**Figure 6:**
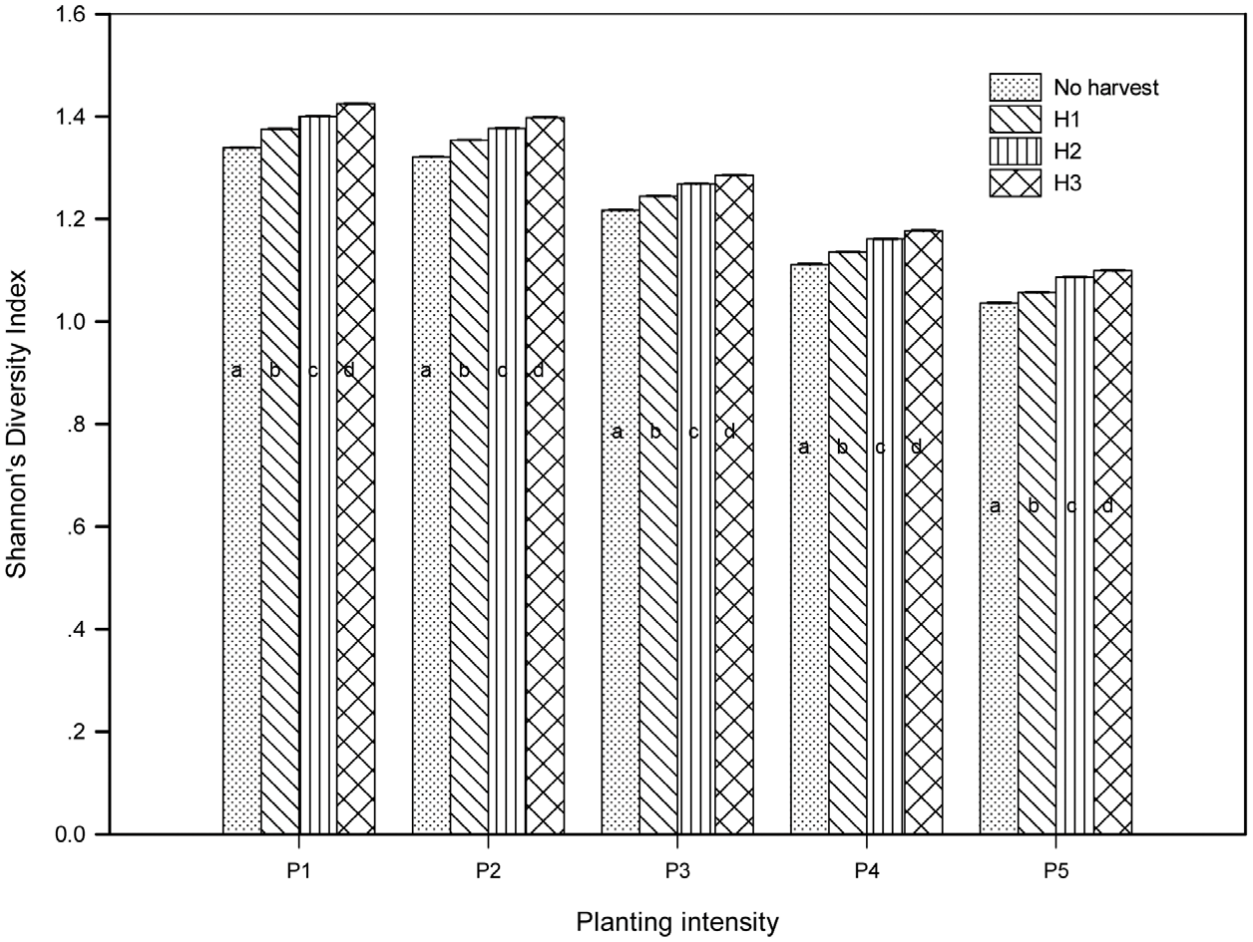
Shannon’s Diversity Index of Hun River upstream forest in simulated scenarios. P1–P5 and H1, H2 and H3: Definitions are given in Table 2; a–d: significant difference (LSD, P<0.01) of Shannon’s Diversity Index within same planting intensity but different harvest intensity.

Shannon’s Diversity Index value showed that increased planting intensity of *P. koraiensis* was followed by decreasing diversity of forest composition, while harvest regimes could slightly increase forest diversity (Fig. 6).

## Discussion

### 1 The Effects of Planting

Planting is an effective method of accelerating forest succession [11], [17], [27], but the most effective and proper planting intensity depends on the forest management strategy and investment budget. We suggest that a 30% planting intensity is the proper planting regime. On one hand, in the long run, it can accelerate the forest to be absolutely dominated by *P. koraiensis* via a more effective rate of planting. On the other hand, it could maintain a higher degree of forest diversity than would be achieved with higher planting intensities such as 50% and 70% (Fig. 6). Our research showed that although the area percentage of *P. koraiensis* at year 300 increased with increasing planting intensity, the sensitivity of response of *P. koraiensis* to planting intensity decreased with increasing intensity (Table 4). Wang et al. [27] also found that species abundance is more sensitive to low intensity planting. The reason for this is because the planted trees occupy the spaces which are probably the living spaces for natural regeneration of trees, then, it turns out that the planting efficiency decreases with the increasing planting intensity. In other words, in a low planting intensity, more space is saved for natural regeneration of trees, so the planting efficiency is higher, but more time is required to increase the coverage of the target species. With a high planting intensity, less space is saved for the seeds of trees, so the planting efficiency is lower, but the time to increase the coverage of the target species is less, because planting trees substitutes for the process from natural regeneration to establishment.

### 2 The Effects of Harvesting

Selective harvest regimes increased the forest diversity, while the planting regimes promoted the homogenization of forest composition (Fig. 6). Moderate disturbance has proven to be a way of increasing ecosystem diversity [39]. Elliott and Knoepp [40] found that there is greater species diversity after harvesting. Hall et al. [41] also found that harvesting could provide a sustainable management strategy for biodiversity conservation. There are two sources of forest diversity, the evenness of abundance of different types species and the number of types of species [42], acording to Shannon’s Diversity Index, but the source of the diversity of our research is the evenness of abundance of different types species. Two reasons for this are: (1) harvesting creates gaps for the establishment of early-successional species [43] and leads to the evenness of abundance of different types of species; (2) the types of species in the model are set according to the vegetation map required for the running of LANDIS, thus there will not be new species migrating into the study area.

The forest diverstity increased with the increasing harvesting intensity (Fig. 6). We propose that the combination of selective harvesting 50% of general timber forest and selectively harvesting 70% of other timber forest could be the choice of harvest intensity, because its negative effect on forest composition is small. For example, *P. davidiana* is the species most sensitive to harvest regimes (Table 6), but even in the scenario P1H3 (Table 2), which is the combination of the lowest planting intensity and the highest harvest intensity, the variability of area percentage of *P. davidiana* is at a low level of around 10% during the simulated 300 years (Fig. 5). This is because harvesting is limited in timber forests (57% of total forest area) and selective harvesting is limited exclusively to the trees at the harvestable ages. Thus while harvest intensity may be high at the patch scale, it is not high at the landscape scale [44].

### 3 The Dynamics of Area Percentage of Some Species

The area percentage of *Q. mongolica* decreased due to the planting of *P. koraiensis,* which is more shade-tolerant than the former, while the dynamics of area percentage of *Q. mongolica* differed from that of other mid-successional species such as *P. tabulaeformis*, *P. densiflora*, *F.chinensis*, *F. rhynchophylla and J. mandshurica* (Fig. 3). We assume this is because: (1) the existing area percentage of *Q. mongolica* exceeds that level typical of a climax forest and as a result there are likely no shade-intolerant species around them; and (2) the planted *P. koraiensis* occupies some of the area that would have been taken up by *Q. mongolica*. Although this *Q. mongolica* forest is not the climax forest for this region and would eventually be replaced by *P. koraiensis* forest according to the history [20], a rapid rate of forest succession toward the *P. koraiensis* forest would not be ideal because climate change appears to be creating a different environment [45], [46].

The early successional species *P. davidiana* showed an abrupt increase in area percentage. In the N and P scenarios, this increase, which occurred around the year 250, would benefit from the death of some species whose longevity is 250 years and establishment efficiency is low, such as *P. sylvestris var. mongolica* and *L. olgensis*. In the PH scenarios, the increase would benefit from the harvesting. Because the seed dispersal ability of *P. davidiana* is strong and the seed can adapt to various environments [47], the gaps which are created by either death of other species or harvesting would have a high probability of being occupied by *P. davidiana*. It is reported that suitable habitat and seed dispersal are key to the distribution and abundance of a species [48], [49]. Due to their high seed dispersal and establishment ability, the early successional species can help restore the ecosystem in case of serious disturbance [49]–[51]. Therefore adequate seed sources for these species should be maintained in the forest.

The area percentage of *L. olgensis* showed a decreasing trend and this species eventually disappeared in this region, because it is shade-intolerant and its establishment ability is low. Although *L. olgensis* is currently distributed widely in the study area, it is all in the form of plantations. The investigation of the vegetation in the mountains of eastern Liaoning province and the predictions of different models in the Changbai Mountain area show that the *L. olgensis* forest is a declining population [52]–[54]. Zhu *et al.* [32] pointed out that *L. olgensis* has difficulty in natural regeneration. In light of the above, we assume that from a management perspective *L. olgensis* is not a proper species for restoration in mid- to climax successional stages.

### 4 Caveats

The dynamics of *Q. mongolica* and *P. davidiana* stimulated in our research merit attention because a trend of higher temperature and less precipitation has been occurring in this region over the last 44 years [55], although the prediction of climate variation is uncertain [56]. (1) *Q. mongolica* is crowed out by *P. koraiensis* in our simulation, but previous research has found that *Q. mongolica* is more resistant to climate warming than *P. koraiensis* [57]. These lead us to recommend the adoption of conservative measures from a management perspective: (a) Promote forest succession via adopting a low planting intensity; (b) Understand and follow the responses of species to the emerging pattern of climate variation, so that management regimes can be altered in time to adapt to these climatic changes. (2) Although the presence of *P. davidiana* would insure restoration after disturbances, it will be a challenge for maintaining ecosystem water balance, especially under climate-induced drought conditions, because consumption of water by early successional species such as *Populus spp.* is high [58]. The pros and cons of harvesting should be carefully evaluated for the abrupt increase of *P. davidiana*.

### Conclusions

With increasing planting intensity the coverage of *P. koraiensis* increases and planting efficiency decreases. This is important information for forest management in the context of limited financial and labor resources. In addition, diversity will decrease with increasing planting intensity. Taking both forest diversity and labor and financial constraints into account, a low planting intensity, such as 30%, in which the forest is restored to the climax forest over the long run, is a better management strategy for restoration.

When timber forests occupy about 50% of the total forest area, an intensity level of selectively harvesting 50% of the general timber forest and selectively harvesting 70% of the remaining timber forest could be appropriate, because the negative effects of this harvest intensity are small at the landscape scale and landscape diversity increases with the increased harvest intensity.

From the dynamic of species, we note two important caveats. (1) Encouraging a rapid pace of forest succession may not be a good management strategy, because the climax species would crowd out other species, some of which would likely be more adapted to future climate conditions in the long run. (2) Careful evaluation of the pros and cons of harvesting is needed. That is because although harvesting can increase forest diversity, we should pay attention to the abrupt increase of early successional species such as *P. davidiana* after harvest, due to their characteristics of high water consumption which will be a challenge for maintaining ecosystem water balance, especially under climate-induced drought conditions.
